# In-vivo-wear in composite and ceramic full mouth rehabilitations over 3 years

**DOI:** 10.1038/s41598-021-93425-z

**Published:** 2021-07-07

**Authors:** Gintare Burian, Kurt Erdelt, Josef Schweiger, Christine Keul, Daniel Edelhoff, Jan-Frederik Güth

**Affiliations:** 1 Department of Prosthetic Dentistry, University Hospital, Ludwig-Maximilians-University Munich, Goethestrasse 70, 80336 Munich, Germany; 2 Department of Prosthodontics, Goethe University, Center for Dentistry and Oral Medicine (Carolinum), Theodor-Stern-Kai 7, 60596 Frankfurt am Main, Germany

**Keywords:** Medical research, Materials science

## Abstract

The aim of this study was to quantify and to compare the wear rates of premolar (PM) and molar (M) restorations of lithium disilicate ceramic (LS2) and an experimental CAD/CAM polymer (COMP) in cases of complex rehabilitations with changes in vertical dimension of occlusion (VDO). Twelve patients with severe tooth wear underwent prosthetic rehabilitation, restoring the VDO with antagonistic occlusal coverage restorations either out of LS2 (n = 6 patients, n = 16 posterior restorations/patient; N = 96 restorations/year) or COMP (n = 6 patients; n = 16 posterior restorations/patient; N = 96 restorations/year). Data was obtained by digitalization of plaster casts with a laboratory scanner at annual recalls (350 ± 86 days; 755 ± 92 days; 1102 ± 97 days). Each annual recall dataset of premolar and molar restorations (N = 192) was overlaid individually with the corresponding baseline dataset using an iterative best-fit method. Mean vertical loss of the occlusal contact areas (OCAs) was calculated for each restoration and recall time. For LS2 restorations, the mean wear rate per month over 1 year was 7.5 ± 3.4 μm (PM), 7.8 ± 2.0 μm (M), over 2 years 3.8 ± 1.6 µm (PM), 4.4 ± 1.5 µm (M), over 3 years 2.8 ± 1.3 µm (PM), 3.4 ± 1.7 µm (M). For COMP restorations, the mean wear rate per month over 1 year was 15.5 ± 8.9 μm (PM), 28.5 ± 20.2 μm (M), over 2 years 9.2 ± 5.9 µm (PM), 16.7 ± 14.9 µm (M), over 3 years 8.6 ± 5.3 µm (PM), 9.5 ± 8.0 µm (M). Three COMP restorations fractured after two years and therefore were not considered in the 3-year results. The wear rates in the LS2 group showed significant differences between premolars and molars restorations (p = 0.041; p = 0.023; p = 0.045). The wear rates in COMP group differed significantly between premolars and molars only in the first two years (p < 0.0001; p = 0.007). COMP restorations show much higher wear rates compared to LS2. The presented results suggest that with increasing time in situ, the monthly wear rates for both materials decreased over time. On the basis of this limited dataset, both LS2 and COMP restorations show reasonable clinical wear rates after 3 years follow-up. Wear of COMP restorations was higher, however prosthodontic treatment was less invasive. LS2 showed less wear, yet tooth preparation was necessary. Clinicians should balance well between necessary preparation invasiveness and long-term occlusal stability in patients with worn dentitions.

## Introduction

The availability of in vivo data on the wear behavior of tooth-colored monolithic dental materials in cases of complete rehabilitation is limited. Loss of dental hard tissue due to tooth wear is a well described clinical problem with an increasing prevalence in dental practice^1, 2^. In general, tooth wear is a result of different mechanisms: interaction with exogenous materials/ three body wear (abrasion), tooth-to-tooth contact/ two body wear (attrition) or chemical dissolution of hard tissues (erosion)^3, 4^. These wear mechanisms often act sequentially or in synchrony, which can lead to accelerated excessive tooth wear at relatively young age. This condition poses several complications as changes in vertical dimension of occlusion (VDO) with possible functional impairment, increased tooth sensitivity and reduced esthetics^4^. At this point severe tooth wear affects people’s quality of life^5^.

The multifactorial conditions of tooth wear implicate restorative challenges for clinicians, as a complex holistic rehabilitation is required, including occlusal adjustment e.g.^6^. New solutions are needed which are in line with state-of-the-art minimally invasive dentistry^7^. Due to CAD/CAM technology, multiple polymers with advantageous features have been introduced to the dental market. These CAD/CAM polymers, manufactured under industrial standards, show superior mechanical properties to those of direct polymers and have even been considered as an alternative to glass–ceramic^8–10^. Multiple advantages of CAD/CAM composites have been already observed in different in vitro studies: a high fatigue resistance, an antagonistic friendly behavior and appropriate optical properties^11, 12^. Consequently, they were implemented in different fields of prosthetic dentistry^13, 14^. Especially in challenging cases of worn dentition, CAD/CAM-fabricated polymer restorations allow to comply with the aims of minimal invasive dentistry and biomimetic approaches^15^.

Both, glass ceramics and composite resins are applied and reported as restorative materials for treatment of severe tooth wear^6, 16, 17^. In respective clinical cases, material thickness plays an important role, as for patients with already advanced hard tissue loss a maximal preservation of the remaining tooth substance is desired^18^. CAD/CAM-based polymer materials are easier and cheaper to manufacture and due to their standardized production show reproducible material properties compared to direct polymer. However, they show inferior mechanical properties, as material hardness and wear resistance, compared to ceramics^19^. Therefore, the question is whether CAD/CAM polymers are worth considering as an appropriate material for long-term prosthetic rehabilitation of severe worn dentition.

Nevertheless, about the wear behavior and wear rates of these dental materials used for treatments of severe worn dentition over a longer period of time, too little is known. In consideration of this limited data availability on in vivo wear performance in cases of complete rehabilitation with increased VDO, the evaluation of wear rates for CAD/CAM polymers and pressed lithium disilicate ceramics, over 3 years, was the aim of the present investigation. A further aim of the study was to assess the differences between wear performance of premolar and molar restorations. The first hypothesis states that restorations made of lithium disilicate ceramic would show lower wear rates than CAD/CAM polymers. Secondly, the wear rates of restorations would be greater in molars than premolars.

## Materials and methods

### Study cohort

This non-blinded, non-randomized study was performed according to the Declaration of Helsinki after approval by the Ethics Committee of Ludwig Maximilian University of Munich (012–12; 541–12). All subjects were informed about the background of the study, the risks associated with it and gave written informed consent before participation in the study.

12 patients (7 males, 5 females, age: mean 36.3 ± 9.4 years) with changes in the vertical dimension of occlusion (VDO) due to severe tooth wear were recruited. Inclusion criteria for this study were as follows: exposed dentin; decreased vertical dimension of occlusion due to loss of dental hard tissue; indication for at least 12 restorations in upper and lower jaws; age between 18 and 70 years; proper oral hygiene; healthy/ treated periodontal tissues (tooth mobility no more than grade I). Exclusion criteria were pregnancy or lactation. Craniomandibular disorders were not explicitly excluded. However, patients underwent a splint therapy previous to the definitive restorative treatment to functionally evaluate the new vertical dimension and bite position.

All patients underwent full-mouth rehabilitation restoring the vertical dimension of occlusion with antagonistic full coverage restorations from the same material. The concept of canine protected centric occlusion could be implemented in every patient.

The patients were divided into two equal groups: group LS2 included 6 patients, who received a full mouth restoration of the upper and lower jaw with adhesively bonded monolithic ceramic restorations [per patient: n = 28, n (premolar) = 8, n (molar) = 8], group COMP consisted of 6 patients, who received a full mouth restoration of the upper and lower jaw with adhesively bonded CAD/CAM restorations [per patient: n = 28, n (premolar) = 8, n (molar) = 8], made of experimental composite blocks, polymerized under standardized industrial conditions.

The wear rates were evaluated using posterior restorations in the maxilla and mandible (LS2: n = 96; COMP: n = 96).

### Treatment and laboratory procedures

In this clinical trial all procedures were performed according to current principles of minimally invasive dentistry. If needed, damaged teeth were restored using direct low-viscosity (Tetric EvoFlow, Ivoclar Vivadent, Schaan, Liechtenstein) and/or high-viscosity composites (Tetric EvoCeram, Ivoclar Vivadent, Schaan, Liechtenstein) and a multi-step adhesive system (Syntac, Ivoclar Vivadent, Schaan, Liechtenstein). Subsequently, tooth preparations were performed according to the material guidelines to receive either LS2 or COMP restorations. The required minimum thickness for restorations made of the lithium disilicate ceramic had been defined as 1.0 mm, the experimental CAD/CAM-composite blanks allowed minimum layer of 0.3 mm in circular area and 1.0 mm in occlusal load bearing zone. Polyether material (Permadyne/Impregum Penta, 3 M, Seefeld, Germany) was used for impressions with individualized rim-lock trays.

Dental restorations were manufactured in a dental laboratory by an experienced dental technician. Press technique was chosen for fabrication of LS2 restorations, instead of CAD/CAM technique, due to similar materials properties and better esthetics. LS2 restorations were fabricated and glazed afterwards. Applied lithium disilicate ceramic (IPS e.max Press, Ivoclar Vivadent, Schaan, Liechtenstein) exhibited following mechanical properties (according to manufacturer): flexural strength = 400 ± 40 MPa, modulus of elasticity = 95 ± 5GPa, Vickers hardness = 5,900 ± 100 MPa. COMP restorations were fabricated using the CEREC system (CEREC InLab V3.86, Dentsply Sirona, Bensheim, Germany). CEREC settings were defined as follows: proximal contact strength = 75 μm, occlusal contacts strength = 25 μm, adhesive gap = 20 μm. Manufacturer reported about composition of the experimental CAD/CAM composite material (Ivoclar Vivadent, Schaan, Liechtenstein), which consisted of 22% V_f_ matrix (dimethacrylate) and 78% V_f_ filler (barium glass fillers, 15%; ytterbium trifluoride, 9%; mixed oxides, 44%; silicon oxides, 3; copolymers, 7%). The material showed mechanical properties as follows: flexural strength = 167 MPa, modulus of elasticity = 11.4GPa, Vickers hardness = 915 MPa and water absorption after 7 days = 28 µg/mm^3^.

If occlusal adjustments were necessary, most could be carried out during the try-in phase, that re-firing or polishing could be done under laboratory conditions. Before luting of the restorations, the following pretreatment methods were applied:

- For the LS2 group: Etching of inner surfaces with 5% hydrofluoric acid (IPS Ceramic Etching Gel, Ivoclar Vivadent, Schann, Liechtenstein) for 20 s, rinsed with air/water spray for 60 s, cleaned in ultrasonic bath for 5 min. 60 s, air dried and silanizated for 60 s using Monobond Plus (Ivoclar Vivadent, Schaan, Liechtenstein).
- For the COMP group: tribochemical silica-coating of the inner surfaces using a modified Rocatec procedure (Rocatec soft 30 µm; 1 bar; nozzle distance: 2 cm; 5 s blast time per unit) and conditioning the restorations with Monobond Plus (Ivoclar Vivadent, Schann, Liechtenstein)

Tooth conditioning for adhesive bonding was identical for both groups, using Total Etch & Rinse technique and Syntac (Ivoclar Vivadent, Schaan, Liechtenstein). Variolink II (Ivoclar Vivadent, Schaan, Liechtenstein) was used as luting composite. Luting curing was performed following manufacturer’s instructions. Variolink II was polymerized for at least 3 times 20 s (vestibular—occlusal, lingual) starting with the proximal margins with a LED curing light [Bluephase Style, Ivoclar Vivadent, Schaan, Liechtenstein (light intensity 1.100mW/cm^2^ ± 10%; wavelength range 385–515 nm)], which was calibrated frequently by a radiometer (Bluephase Meter II, Ivoclar Vivadent, Schaan, Liechtenstein). If occlusal corrections were necessary after placement, restorations were carried out with corresponding diamond burs and polishers (ball shaped diamond finishing bur: 8801 314 018; polishing sets: Composite: Set 4312A, Ceramic: 4313B; Komet Dental, Lemgo, Germany). No occlusal splint was applied after the final treatment.

### Data acquisition of baseline and follow-up records

To acquire baseline and follow-up data, dental impressions with a polyether impressions material (Impregum Penta; 3 M, Seefeld, Germany) were taken after placement of restorations (baseline) and annual recall appointments (follow-up). Mean monitoring time in both groups was 350 ± 86 days (1st year), 755 ± 92 days (2nd year), 1102 ± 97 days (3rd year). All impressions were casted between 24 and 48 h with type 4 dental stone (Plurastone; Pluradent, Offenbach, Germany). Resulting plaster casts were digitalized with a laboratory scanner (D810; 3Shape, Copenhagen, Denmark) and exported to standard tessellation language (STL) file format.

### Processing of datasets

**Figure 1:**
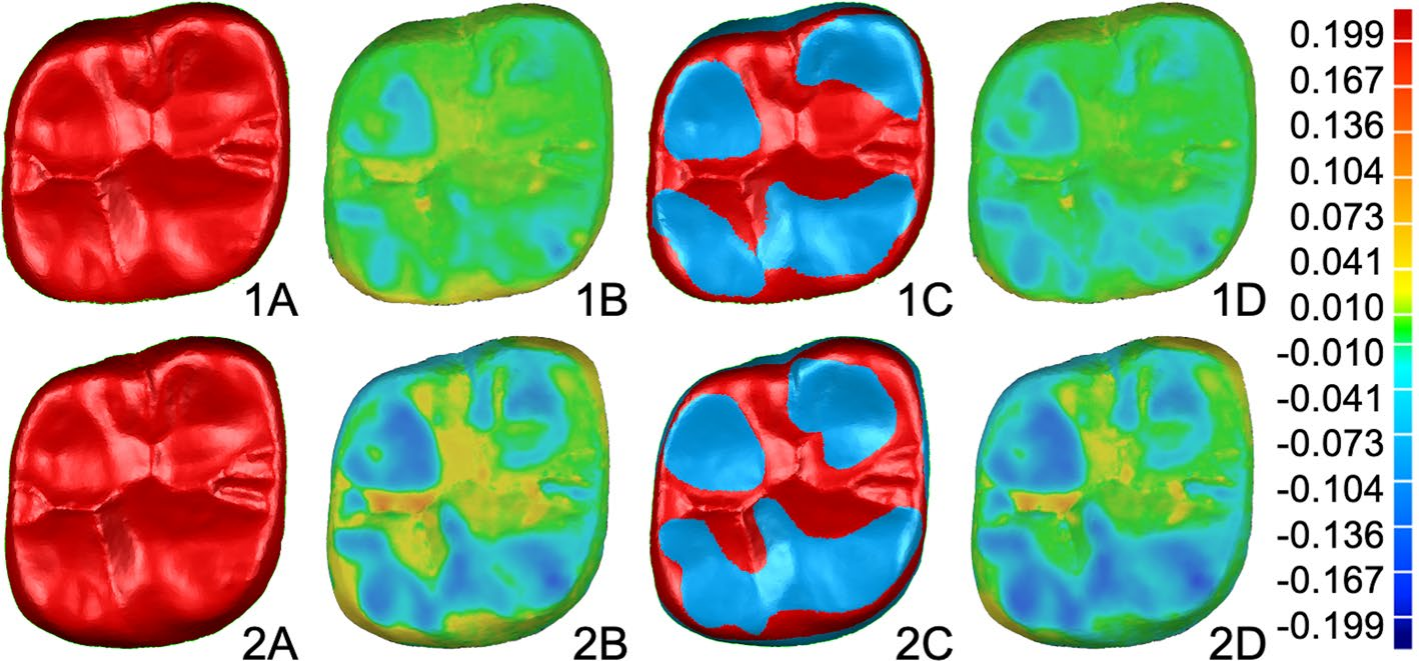
Color-coded superimposition procedure and the wear progression of first molar CAD/CAM composite restoration (Patient 6) in the first (1) and third (2) year. (**1A**/**2A**) pre-alignment over the full occlusal area; (**1B**/**2B**) differences between baseline and recall dataset after the pre-alignment; (**1C**/**2C**) iterative selection of areas, which were not exposed to wear, for further alignment of the data; (**1D**/**2D**) visualization of wear areas after the final data alignment.

Geomagic Qualify software (2012.1.2, Geomagic Inc., Morrisville, NC, USA) was used to compare resulting baseline and follow-up STL datasets. To receive independent data of each specimen every posterior restoration was segmented and stored as individual dataset, resulting in 192 (LS2 n = 96; COMP n = 96) baseline and 570 (LS2 n = 288; COMP n = 282) follow-up records. Three molar restorations in COMP group (patient 5) were excluded from further analysis due to mechanical failure of restorations after two years. Every recall dataset was superimposed with the equivalent baseline record. At the beginning the entire surface matching was performed using best-fit method, resulting superimposition was visually estimated and next best-fit matching was executed only over surface areas with no signs of wear (Fig. 1). This iterative procedure was conducted until superimposition error remained stable. The overlay error of each specimen was listed separately. A superimposition error of more than 15 µm was determined as exclusion criteria for further data processing, as the influence of impressioning, model fabrication, scanning, and superimposition was considered too high for reliable data analysis. Due to this, the amount of data in LS2 group was reduced from 288 to 279, in COMP group from 282 to 258. It must be noted that out of 33 data sets, which dropped out, 8 were premolar and 25 molar restorations (Table 1).

**Table 1:** Mean values ± standard deviation (µm) of superimposition error between baseline und follow-up datasets.

Material	Time [year]	n	Premolar	n	Molar
LS2	1	46	6.8 ± 1.8	45	8.3 ± 1.8
	2	48	7.5 ± 1.9	47	9.1 ± 1.7
	3	47	8.2 ± 2.1	46	10.9 ± 2.1
COMP	1	47	9.9 ± 2.1	48	11.7 ± 2.5
	2	47	10.8 ± 2.1	39	12.0 ± 2.2
	3	45	11.9 ± 2.3	32	13.0 ± 1.7

Subsequently to the overlay procedure, remained datasets underwent wear calculation perpendicular to the surface of the restoration, results were exported and saved separately for each specimen as a result record (.csv). Calculation of mean wear rates per month was chosen for further analysis.

### Statistical analyses

The obtained data was transferred into a statistical program SPSS (version 25, IBM, Armonk, NY, USA) for further processing. The figures were generated using GraphPad Prism **(**version 8.4.2., GraphPad Software Inc., La Jolla, CA, USA). To guarantee best possible comparability of our results despite varying recall appointments and performed data filtering due to superimposition error, wear values were computed by dividing the results by the number of days in situ. Subsequently, the mean wear rate per month was calculated for both groups. Normality of data distribution of all groups was analyzed using Kolmogorov–Smirnov test. The Kruskal–Wallis test was used to analyze data interaction between variables, following the Wilcoxon signed-rank test or pairwise comparison with Mann–Whitney U test, which were applied to evaluate statistically significant differences between groups. In cases of multiple comparisons Bonferroni correction was used. The significance level was set at 0.05. The mean and median vertical loss was calculated, and outliers were eliminated by SPSS software. This elimination implicated decrease of follow-up data sets in both groups, in LS2 group from 279 to 253, in COMP group from 257 to 241.

## Results

### Measurements of superimposition error

**Figure 2:**
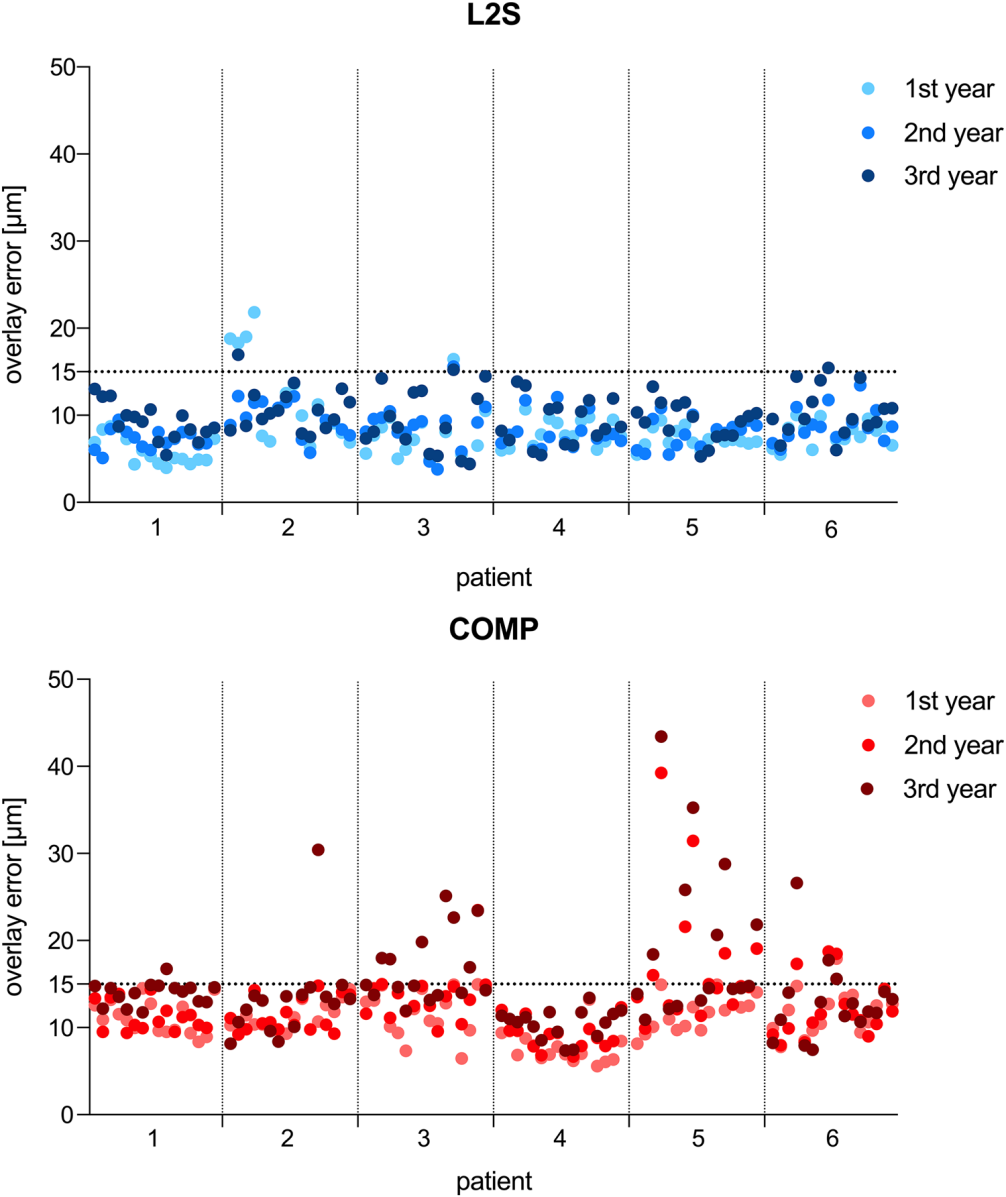
Visualized superimposition error (µm) of each specimen and individual patient. Superimposition error under 15 µm was defined as the requirement for further data analysis.

Mean superimposition error was calculated according to material, time and tooth group. Differences between mean values were found regarding every characteristic. The COMP group showed enlarged errors trough matching process compared to the LS2 group. Premolars were easier to superimpose than molars in both groups. The differences regarding follow-up time were found. Higher superimposition errors were found with advancing age of restauration in situ (Table 1; Fig. 2).

### Measurements of wear rates

**Figure 3:**
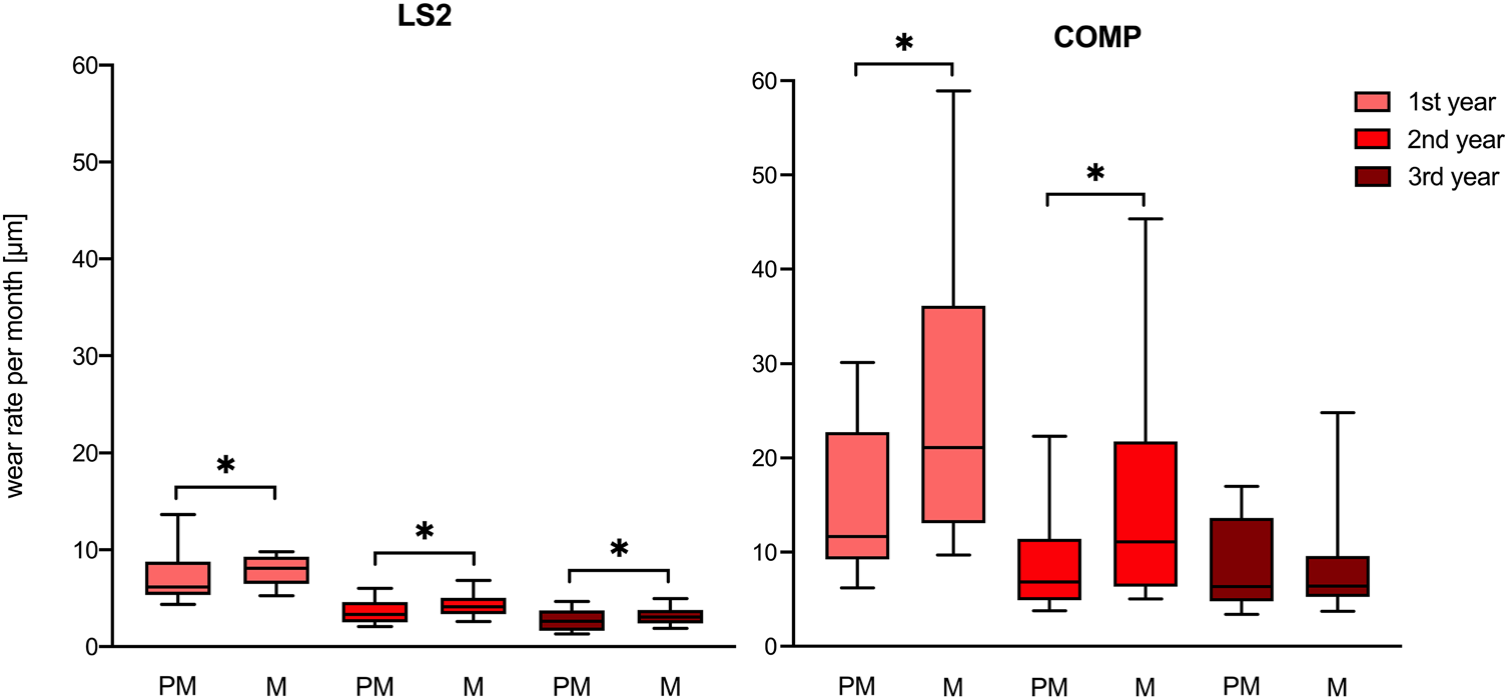
Boxplots illustrate median and IQR values of wear rates (μm/month) for premolar (PM) and molar (M) restorations. Comparison within teeth group (premolar vs molar)—Mann–Whitney U test [*Statistically significant differences].

75% of data groups were not normally distributed. Therefore, non-parametric tests were applied to evaluate differences between the test groups. Calculated wear rates are shown in Tables 2 and 3 . Boxplots are given in Fig. 3. Wear rates decreased over time, therefore significant differences were found between different time intervals (LS2 PM 1–2 years p < 0.0001, 1–3 years p < 0.0001, 2–3 years p = 0.033; LS2 M 1–2 years p < 0.0001, 1–3 years p < 0.0001, 2–3 years p = 0.013; COMP PM 1–2 years p < 0.0001, 1–3 years p < 0.001, COMP M 1–2 years p = 0.002, 1–3 years p < 0.0001; 2–3 years  = 0.037). No differences in PM wear rates of 2–3 years interval in COMP group were detected (p = 1.000). When comparing the premolar and molar wear rates significant differences in LS2 group were identified (1 year p = 0.041; 2 years p = 0.023; 3 years p = 0.045). The premolar and molar wear rates in COMP groups showed significant differences during one and two years of observation (1 years p = 0.0001; 2 years p = 0.007), over 3 years no significant differences were found (3 years p = 0. 862). Statistical comparison of wear rates between LS2 and COMP showed significant differences (1 years years: PM p < 0.0001, M p < 0.0001; 2 years: PM p < 0.0001, M < 0.0001; 3 years: PM p < 0.0001, M < 0.0001).

**Table 2:** Mean values ± standard deviation (µm/month) of wear rates of premolar and molar restorations for individual patient, respectively.

	Patient	Sex	Age [year]	Time [year]	n	Premolar	n	Molar
*LS2*	1	Female	37	1	7	6.7 ± 2.1	7	6.7 ± 1.3
				2	8	5.3 ± 1.7	7	3.8 ± 0.5
				3	8	3.3 ± 1.3	8	2.8 ± 0.9
	2	Female	40	1	4	10.4 ± 5.1	5	9.3 ± 1.0
				2	7	5.2 ± 1.4	8	4.7 ± 1.2
				3	7	4.2 ± 1.6	6	3.9 ± 1.0
	3	Male	32	1	7	5.4 ± 0.7	7	8.2 ± 2.7
				2	7	3.3 ± 1.4	7	5.3 ± 1.3
				3	8	2.2 ± 0.9	6	3.2 ± 0.5
	4	Male	40	1	8	8.9 ± 2.9	6	8.6 ± 5.5
				2	6	3.2 ± 1.0	8	3.3 ± 0.9
				3	7	2.2 ± 0.5	7	2.8 ± 0.7
	5	Male	20	1	7	5.1 ± 1.1	7	6.4 ± 2.3
				2	8	2.6 ± 0.7	8	4.7 ± 1.4
				3	6	2.0 ± 0.7	8	2.8 ± 0.8
	6	Male	31	1	6	9.6 ± 5.0	7	7.9 ± 1.7
				2	8	3.1 ± 0.8	8	4.7 ± 2.3
				3	7	2.7 ± 1.2	7	5.1 ± 3.3
*COMP*	1	Female	44	1	7	11.7 ± 3.3	8	12.4 ± 5.5
				2	8	6.8 ± 2.4	8	5.5 ± 1.5
				3	7	6.4 ± 2.3	8	5.0 ± 1.3
	2	Female	38	1	7	7.9 ± 1.8	7	12.8 ± 3.3
				2	8	4.6 ± 1.4	7	8.4 ± 2.1
				3	7	3.6 ± 0.8	6	5.7 ± 1.8
	3	Female	38	1	7	13.8 ± 8.9	8	48.2 ± 21.6
				2	7	11.0 ± 6.8	7	40.4 ± 16.6
				3	6	11.8 ± 4.2	2	31.6 ± 7.2
	4	Male	51	1	6	10.9 ± 1.0	8	20.7 ± 9.8
				2	8	6.2 ± 1.2	8	13.0 ± 5.5
				3	7	5.2 ± 0.8	8	6.7 ± 2.3
	5	Male	47	1	7	28.0 ± 7.0	8	47.8 ± 22.8
				2	8	18.1 ± 5.9	2	28.1 ± 3.1
				3	7	17.6 ± 2.3	1	25.9
	6	Male	18	1	7	19.9 ± 8.0	8	26.8 ± 9.0
				2	7	8.9 ± 3.0	4	13.5 ± 1.7
				3	6	7.2 ± 1.0	6	13.0 ± 6.0

**Table 3:** Wear rates (µm/year) for premolar and molar restorations.

Material	Time [year]	n	Premolar		n	Molar	
			Mean (SD)	Median (IQR)		Mean (SD)	Median (IQR)
*LS2*	1	39	90 (40.8)	73.2 (40.8)	39	93.6 (24)	97.2 (33.6)
	2	44	45.6 (19.2)	39.6 (25.2)	46	52.8 (18)	49.2 (20.4)
	3	43	33.6 (15.6)	31.2 (25.2)	42	40.8 (20.4)	37.2 (16.8)
*COMP*	1	41	186 (107)	140 (162)	47	342 (242)	253 (276)
	2	46	110 (70.8)	81.6 (78.1)	36	200 (179)	133 (185)
	3	40	103 (63.6)	75.6 (105)	31	114 (96)	76.8 (51.6)

## Discussion

The aim of this clinical study was to compare different materials’ wear rates in patients after full mouth prosthetic rehabilitation with full occlusal coverage restorations due to severe tooth wear. Additional target was to compare differences between premolar and molar wear rates regarding the materials used. The first hypothesis was confirmed, as the wear rates of CAD/CAM composite restorations were significant higher compared to lithium disilicate. The second hypothesis must be partly rejected. The CAD/CAM composite group showed increased wear in the molar region in first two years. At 3 years, no significant differences were found, however this might be due to the fact that fewer restorations could be included after 3 years. Restorations made of lithium disilicate ceramic showed significant differences between premolar and molar wear rates with advancing age of restorations in situ. Much higher wear rates in both groups over first year confirm specific running-in wear in initial phase after placement of restorations^20^.

Wear is considered as a dynamic and multilayered process, which emerges by surfaces interaction with exogenous materials and/or under teeth contact, what results in continuous loss of the tooth structure or restorative material^21^. Nevertheless, different restorative materials show different wear mechanisms. While ceramic wear occurs by progressing microfracture process, composite restorations abrade by adhesion wear, which follows plastic deformation of the material^22, 23^. Multiple in vitro investigations on lithium disilicate ceramics, as well as on CAD/CAM composites, provided clinical acceptable mechanical properties with tolerable wear behavior on opposing enamel^10, 12, 24^. However, the used methods for in vitro wear quantification differ from methods used in clinical trials, which complicates comparability^25, 26^. Therefore, it is of great necessity to perform in vivo studies on materials wear performance, especially in cases of complete occlusal treatment with bite elevation. The primary goals of these complex therapies are to improve aesthetics, prevent further hard tissue loss and reestablish functional occlusion. However, the main focus in treatment with raise in VDO should always remain in creating harmony in the masticatory system through stable occlusion, which is important for long-term results. In line with this, material wear behavior in these cases plays a major role, as any disharmony in wear may result in adaptive responses like increased functional activity, which results in excessive wear of restorations. Therefore, there is a need to study occlusal wear in cases of full mouth rehabilitations. On the contrary, all clinical trials face numerous challenges, as patient recruitment and retention, funding, and possible clinical complications.

To date, different opinions on VDO alteration and possible consequences exist in the literature^27^. Several authors described possible complications during implementation of bite elevation, like increase of bite forces, hyperactivity of masticatory muscles, temporomandibular disorders and phonetic limitations^28–31^. At the same time, numerous case reports of successful treatment of severe worn dentition with VDO alteration are published in literature^32, 33^. This comes to the point, that the increased restorations wear was not considered or defined as a potential complication, which unfortunately is difficult to assess through clinical examination.

The biomimetic approach in last decade inspired clinicians and scientists to search and to implement restorative materials with comparable wear behavior as of natural tooth enamel^34, 35^. Lambrechts et al.^36^ determined enamel wear as 15 μm/year for premolars and 29 μm/year for molars, which is still held as clinical standard for comparison until this day. Clinical studies on wear of lithium disilicate glazed press core ceramic with opposing enamel reported mean vertical loss between 30 and 40 μm per year^37, 38^, which does not differ much from findings of full coverage composite crowns with values around 44 μm per year^39^. However, these results are ascertained in functional harmonious occlusion, where a maximum of three restorations were placed. In this context it may be suggested that the crowns may be protected by the adjacent structures, which resulted in comparable wear rates to enamel. So one could conclude, if composite restorations are protected by adjacent teeth and established anterior/canine guidance, composite might be the alternative of choice if the antagonist is enamel. This implication further complicates possible comparison with obtained data in this study, as results show strong distinction, with wear rates of lithium disilicate ceramic around 90 μm in first year, with significant differences between premolar and molar restorations. In contrast to these results, CAD/CAM polymer shows significant higher wear rates, with a mean vertical loss of 186 μm in premolar region, and 342 μm in molar region during the first year. However, it must be noted, that in this study, full occlusal load had to be absorbed by the tested restorations. Therefore, use of occlusal splint could be recommended for minimizing the wear progression.

It can be assumed, that some patients possibly showed increased functional activity, which not only resulted in higher restorations wear, but also showed significant differences in material wear (Table 2). Varieties of wear rates of the posterior region can also reflect varying masticatory forces from tooth to tooth, accordingly to Ferrario et al.^40^. This could explain increased wear on molar restorations in the CAD/CAM polymer group. The differing mechanical properties compared to lithium disilicate ceramic might lead to the observed wear differences between molars and premolars. However, for interpretation, one must keep in mind, that every imbalance in occlusion, could be adjusted by dentoalveolar compensation through possible extrusion of worn teeth^41, 42^.

When interpreting the results of this study, several methodical limitations need to be considered. The limitations of the workflow and process accuracy (impression, casting, scanning) that lead to the underlying data needs to be mentioned and are intensively discussed in a previous article applying the same methodology^43^. Also, the data of experimental CAD/CAM composite should be treated with caution due to its composition and properties. Therefore, it is of importance to mention that the findings of this study might not be transferrable to other CAD/CAM composites that are accessible on the dental market with varying characteristics, which can lead to differing wear rates as assessed in this clinical study. This study had a comparable but small patient cohort, which confined possible investigation of age and gender dependency correlation on wear. Secondly, the evaluation of masticatory bite forces and actual VDO increase would have allowed to provide an insight in potential interdependency with the abrasion of the materials. Thirdly, although, the comparatively high standard deviations of our data are inevitable, it is impossible to build homogenous patient groups regarding individual wear patterns and Table 2 shows these inherent differences of patients. Additionally, ongoing vertical loss of restorations over time does not represent a linear progression with constant rate, but rather a logarithmic one. In this regard, it can be concluded that the measured wear rates per month are not a constant value, as well as wear rates per year. Further, we decided to use press technique, instead of CAD/CAM, for fabrication of ceramic restorations, due to similar mechanical characteristics however slightly better optical properties^44^. Lastly, it’s not possible to evaluate total wear of the restorations over the years, due to the loss of follow-up data after filtering procedure, especially of those patients, who showed higher wear rates in the composite group. This loss of the data is due to formation of enlarged occlusal contact areas and possible deformation if restorations over time, which complicates superimposition procedure as only surface areas with no changes and signs of wear can be used.

Complex rehabilitation with alteration of vertical dimension of occlusion (VDO) remains a major challenge for clinicians. Restorations made of lithium disilicate ceramic show more stable and evenly distributed wear rates compared to CAD/CAM polymer in cases with VDO alignment. Nevertheless, there is still a need for longitudinal clinical studies with larger cohorts, to provide better prediction on wear behavior of dental materials and to clarify clinical outcomes of used treatments as well as reference data of natural teeth. Decoding patient’s individual abrasion pattern would allow more personalized dentistry by selecting the most suitable material for a complex rehabilitation. The biggest concern is that despite all the recent innovations and considerable advantages of indirect composite resins, their suitability for complex restorative treatments of generalized tooth wear might be questionable in the long run, considering the wear behavior especially in the load bearing zone.

## Conclusion

Significant differences in COMP group in first two years between premolar and molar wear rates were assessed. Significant differences of premolar and molar wear rates in LS2 group were found, respectively, in the yearly investigations. Wear of COMP restorations was higher, than LS2, however the restorative procedure was less invasive. Clinicians should balance well between necessary preparation invasiveness and long-term occlusal stability in patients with worn dentition.
